# Deep cervical lymph node analysis in central nervous system inflammatory disease

**DOI:** 10.3389/fimmu.2026.1747114

**Published:** 2026-01-26

**Authors:** Alexis Elena Giff, Mattia Wruble Clark, Shamik Bhattacharyya, Peter T. Sage, Bruno Madore, Jeffrey P. Guenette, Edison K. Miyawaki

**Affiliations:** 1 Department of Neurology, Harvard Medical School, Boston, MA, United States; 2 Department of Neurology, Mass General Brigham/Brigham and Women’s Hospital, Boston, MA, United States; 3 Renal Division, Mass General Brigham/Brigham and Women’s Hospital, Transplant Research Center, Boston, MA, United States; 4 Department of Radiology, Mass General Brigham/Brigham and Women’s Hospital, Boston, MA, United States

**Keywords:** cervical lymph nodes, glymphatic, lymphatic, meningeal lymphatic vessels, MRI, neuroimmune, neuroinflammation

## Abstract

A previously espoused notion that the brain is an immune-privileged organ has been challenged by evidence of bidirectional communication between the central nervous system and the periphery. A well-described “glymphatic” system in the brain and the meningeal lymphatic system serve as conduits through which antigens, immune cells, and metabolic waste travel from the brain to the deep cervical lymph nodes. These nodes, which are more than passive drainage points, serve as locales where dendritic cells, T cells, and B cells interact with central nervous system-derived signals and modulate immune responses that can influence the brain itself. Disruption of clearance mechanisms to deep cervical nodes—due to intracranial vascular disease, aging, poor sleep, chronic inflammation, or other etiologies—may lead to immune dysregulation. Abnormalities in lymphatic drainage can also alter the presentation of antigens from the central nervous system, affect lymphocyte trafficking, and contribute to the aggregation of proteins like β-amyloid, tau, and α-synuclein. This review synthesizes current knowledge on glymphatic and meningeal lymphatic anatomy and function, highlights how impaired drainage contributes to disorders including multiple sclerosis, Alzheimer disease, and Parkinson disease, and discusses the emerging role of deep cervical lymph node imaging and immunophenotyping in assessing neuroinflammation. Finally, we consider how modulation of meningeal lymphatic and nodal function, through pharmacologic or physical interventions, may impair or restore drainage and alter the course of disease in various ways. The integration of advanced imaging with immunological analysis ultimately may enhance the diagnosis, monitoring, and treatment of neuroinflammatory and neurodegenerative diseases. We propose that deep cervical lymph nodes represent an understudied locale, and, potentially, a therapeutic target for peripheral interventions to influence brain disease trajectories.

## 1 Introduction

Inflammatory diseases of the central nervous system (CNS) remain enigmatic, largely because we have an incomplete understanding of how immune surveillance is coordinated between the brain and peripheral immune tissues. Recent work has challenged the notion that the brain is immune-privileged, introducing mechanisms by which antigens, immune cells, and waste traffic *bidirectionally* between the CNS and peripheral immune tissues. Bridging the brain and periphery are the glymphatic and meningeal lymphatic networks. A recently elucidated “glymphatic” system clears cerebrospinal fluid (CSF) and brain-parenchyma-derived solutes. This efflux subsequently enters meningeal lymphatic vessels and, to a lesser extent in humans than in other animals, nasal mucosal lymphatics and other drainage pathways. The meningeal lymphatic system is recognized as a pathway not just for waste movement, but also for the egress of immune cells from the central nervous system, after which these cells can function as messengers to the periphery. Much of this waste ultimately drains to the cervical lymph nodes. The deep cervical lymph nodes (dcLNs) are of particular interest due to their ability to respond to CNS antigens and mount responses *within* the CNS (1–4). The so-called efferent arm of the immune response also exists whereby lymphocytes can migrate from the periphery into the CNS and influence brain function (3, 5–7). Disruption along these pathways can occur because of inflammatory signals, aging, poor sleep quality, and alterations in vascular health, among other factors.

In this review, we synthesize current knowledge on the anatomy, physiology, and immune functions of the glymphatic system, meningeal lymphatic system, and other exit routes, as well as highlight how cervical lymph node analysis might help to reshape neuroinflammatory disease management. Our interest in lymph node analysis, however, is exploratory, and cannot currently inform therapeutic decision making in patients. Nevertheless, we draw from emerging evidence in animal models as well as in humans to demonstrate how the use of imaging, immunophenotyping, and targeted interventions could potentially influence clinical decision making in human neuroinflammatory processes.

## 2 The lymphatic and glymphatic systems

In most tissues of the body, lymphatic vessels are the primary means by which excess fluid, proteins, and other metabolic by-products are eliminated. Since the brain’s parenchyma is devoid of conventional lymphatic vessels, waste was believed to diffuse passively into CSF-filled ventricles (8). In 2012, however, two-photon imaging of anesthetized mice revealed that the brain has an intricate system of waste clearance (9). Rather than a traditional lymphatic system, the CNS relies on a highly organized clearance mechanism that operates through glial water channels—the glymphatic system (10). This system has since been demonstrated in both mice and humans.

The glymphatic system channels CSF through specialized spaces surrounding blood vessels, called perivascular spaces. As arteries and veins course from the subarachnoid space into the brain’s parenchyma, they are first enclosed by an extension of the pia mater, forming CSF-filled perivascular spaces, known as Virchow-Robin spaces (11). These spaces around penetrating vessels are bordered externally by astrocytic endfeet. Astrocytic endfeet regulate the blood-brain barrier (BBB) and facilitate the exchange of substances between blood and brain parenchyma. As vessels venture further into the parenchyma, the perivascular space progressively narrows and is no longer discernible at the capillary level. Instead, it tightens into a basal lamina, which is porous and minimally resistant to CSF flow (11). The entry of CSF into the parenchyma is mediated directly by aquaporin-4 (AQP4) channels expressed on the astrocytic endfeet of the perivascular spaces; the channels are influenced by noradrenergic tone: reduced adrenergic signaling during sleep enhances glymphatic drainage (9, 12).

**Figure 1:**
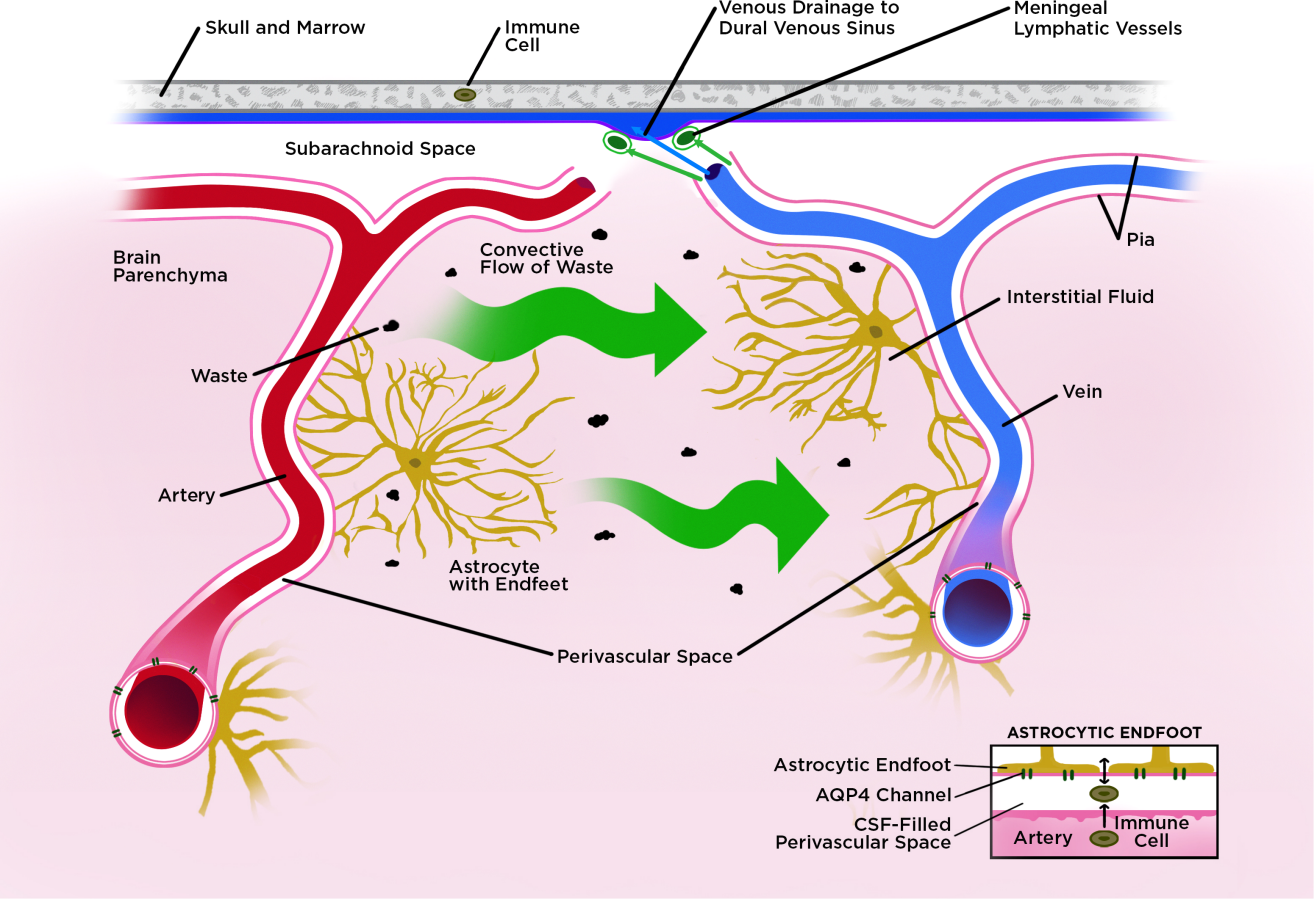
Schematic diagram of glymphatic flux and drainage into meningeal lymph vessels. Structures are not drawn to scale. The glymphatic system operates via specialized spaces surrounding blood vessels called perivascular spaces (also known as Virchow-Robin spaces). As arteries and veins course from the subarachnoid space into the brain parenchyma, they are enveloped by pia mater, forming these perivascular compartments. *Both* arterial and venous perivascular spaces are externally bordered by astrocytic endfeet, which contribute to the blood–brain barrier (BBB) and mediate bidirectional exchange between the vasculature and the brain parenchyma (we depict only the arterial perivascular space in the inset). CSF that has exited the arterial perivascular space through AQP4 channels mixes with intraparenchymal interstitial fluid (ISF), which contains diverse waste products. This mixing generates a directed flow, known as a vectorial convective flux, toward the perivenous space. Once in the perivenous space, waste is cleared via the meningeal lymphatic vessels as well as other pathways that are still being elucidated.

CSF that has exited the perivascular space through AQP4 channels mixes with intraparenchymal interstitial fluid (ISF), which contains diverse waste products (8). This mixing generates a directed flow, known as a vectorial convective flux (**Figure 1**). The movement of CSF and ISF depends largely on cerebral arterial pulsation. Mouse studies in particular have found that modifying blood pressure can increase backflow and reduce net flow in perivascular space (13), while ligating the carotid artery can reduce pulsatility and slow the rate of CSF-ISF exchange (14). This directed flow leaves the parenchyma through AQP4 channels via the astrocytic endfeet of perivenous spaces (9, 15). Tracer studies in rodents have demonstrated that 40-80% of large proteins and other solutes in extracellular space can be removed by vectorial convective flux (9).

As waste is conveyed along the perivenous spaces, it may exit the brain through several pathways, many of which remain the subject of ongoing investigation in animal studies (16–18). These routes include exit via skull foramina along cranial nerves; transport through the cribriform plate to the lymphatic channels of the nasal mucosa (19, 20); egress via dural lymphatics that course alongside the dural venous sinuses and middle meningeal arteries; and passage through the arachnoid villi of the dural venous sinuses for absorption into the bloodstream. The last possibility is still debated in humans, due to the lack of arachnoid granulations in childhood and in some adults (21, 22). Transit may also occur through the calvarial bone marrow via dura-skull channels, after which the CSF’s fate is undetermined (23, 24). Animal studies suggest that roughly 50% of CSF tracers drain into extracranial lymph, with most of the rest entering the bloodstream (25–27). Eventually, waste products transported through the lymphatic system and bloodstream converge in the liver, where they are degraded.

Several factors can influence CSF flow and fluid flux from brain to the periphery via the glymphatic system, which is predominantly active at night (28). The first, and most important, is sleep. The interstitial space, where the CSF-ISF exchange or interchange occurs, enlarges by 60% during sleep. CSF flux is the highest during slow-wave sleep in mice, while studies in humans show that sleep deprivation impairs molecular clearance (29, 30). During slow-wave deep sleep, there is a reduction in CNS noradrenergic tone and an increase in the ISF volume fraction (31). A cocktail of norepinephrine receptor antagonists in awake mice increases CSF influx almost to the level seen in sleeping mice (30). Glymphatic function is also highly active in anesthetized mice, indicating that sleep versus wakefulness, not circadian rhythm *per se*, regulates egress via the glymphatic system (11, 32). Finally, the lateral sleep position is better for glymphatic clearance as compared to the supine or prone positions in mice, and very early work suggests the same may hold true for humans (28, 33). The dependence of glymphatic clearance on modifiable factors like sleep, body position during sleep, and vascular health suggests that many neurodegenerative diseases could be at least in part affected by optimization of these physiological parameters.

Clearance through the glymphatic system slows with age. Contributing mechanisms include decreased vessel-wall pulsatility, loss of AQP4 channel expression, and altered polarization of AQP4 along astrocytic endfeet (34, 35). A decrease of up to 80-90% in glymphatic activity has been observed in old compared to young mice (11). AQP4 channels upregulate with sleep fragmentation in younger animals but downregulate in older animals (36).

Other modifiable factors have been shown to impact glymphatic clearance in mice as well: omega-3 polyunsaturated fatty acids, physical activity, and small amounts of alcohol exert a positive impact, while chronic stress and arterial hypertension exert a negative effect (37). Both acute and chronic inflammation, whether from systemic insults or within the CNS itself, can impact glymphatic function and CSF flow. Solutes from the glymphatic system ultimately exit along various lymphatic routes, which we discuss next.

### 2.1 Lymphatic pathways connecting the CNS to the periphery

Broadly, there are two types of peripheral lymphatic vessels: initial and collecting vessels. ISF first enters the lymphatic system via the initial lymphatics, which are highly permeable and facilitate unidirectional influx of fluid and solutes. Fluid then flows from the initial lymphatics to the deeper collecting vessels, which contain smooth muscle and lymphatic valves (38, 39).

The meningeal lymphatic vessels (mLVs) are most like “initial” lymphatics, acting as conduits for fluid and immune cell entry. These vessels are one of two routes by which lymphatic drainage occurs (based on CSF tracer studies in mice); the other route occurs via the nasal mucosa lymphatics (19, 39, 40). Both systems predominantly drain to the dcLNs. Evidence of the nasal mucosal drainage mechanism is not as consistent in humans, but meningeal lymphatics have an established presence in humans and they play a role in trafficking immune cells (41, 42). Studies in various animals have suggested that ~50% of CSF is drained through mLVs, with the remaining 50% draining from the spinal cord to the mediastinal, iliac, and sacral lymph nodes (43). Drainage through mLVs then ends in the dcLNs. As will be discussed further, research has suggested that dysfunctional mLV drainage contributes to the development or evolution of various CNS pathologies (43).

The mLVs mostly course alongside the dural venous sinuses and middle meningeal arteries and are separated from CSF in the subarachnoid space (**Figure 1**). The mLVs can absorb CSF from the subarachnoid space and glymphatic system. First delineated in 1787 by Giovanni Paolo Mascagni, meningeal lymphatics were only recently characterized across species: rodents (19, 39), zebrafish (44, 45), primates, and humans (39, 46). Recent literature has categorized intracranial mLVs based on their location and function into two groups: dorsal mLVs, which are found in the dorsal skull and have been the most thoroughly explored, and basal mLVs, which are found at the base of the skull and remain less investigated (43, 47). Dorsal mLVs are characterized as morphologically immature with small diameters. Both dorsal and basal mLVs lack smooth muscle cells. The precise contribution of each type of mLV and the significance of certain structural features, such as the proximity of some basal mLVs to skull foramina, have yet to be fully elucidated (43).

The nasopharyngeal lymphatic plexus (NPLP) is the other main CSF drainage route that has been elucidated in mice and macaque monkeys, but appears to play a less significant role in humans (41, 48). The plexus empties into upper (submandibular, superficial and upper jugular cervical lymph nodes) (see anatomy discussion below), with some drainage to retropharyngeal nodes as well (49). The NPLP in mice and non-human primates drains some CSF from mLVs to the dcLNs, perhaps through the medial and lateral deep cervical lymphatic vessels (43). Since the significance of the NPLP is still uncertain in humans, we will focus on mLVs, which are clearly involved in human CSF drainage to the dcLNs.

### 2.2 Deep cervical lymph nodes

**Figure 2:**
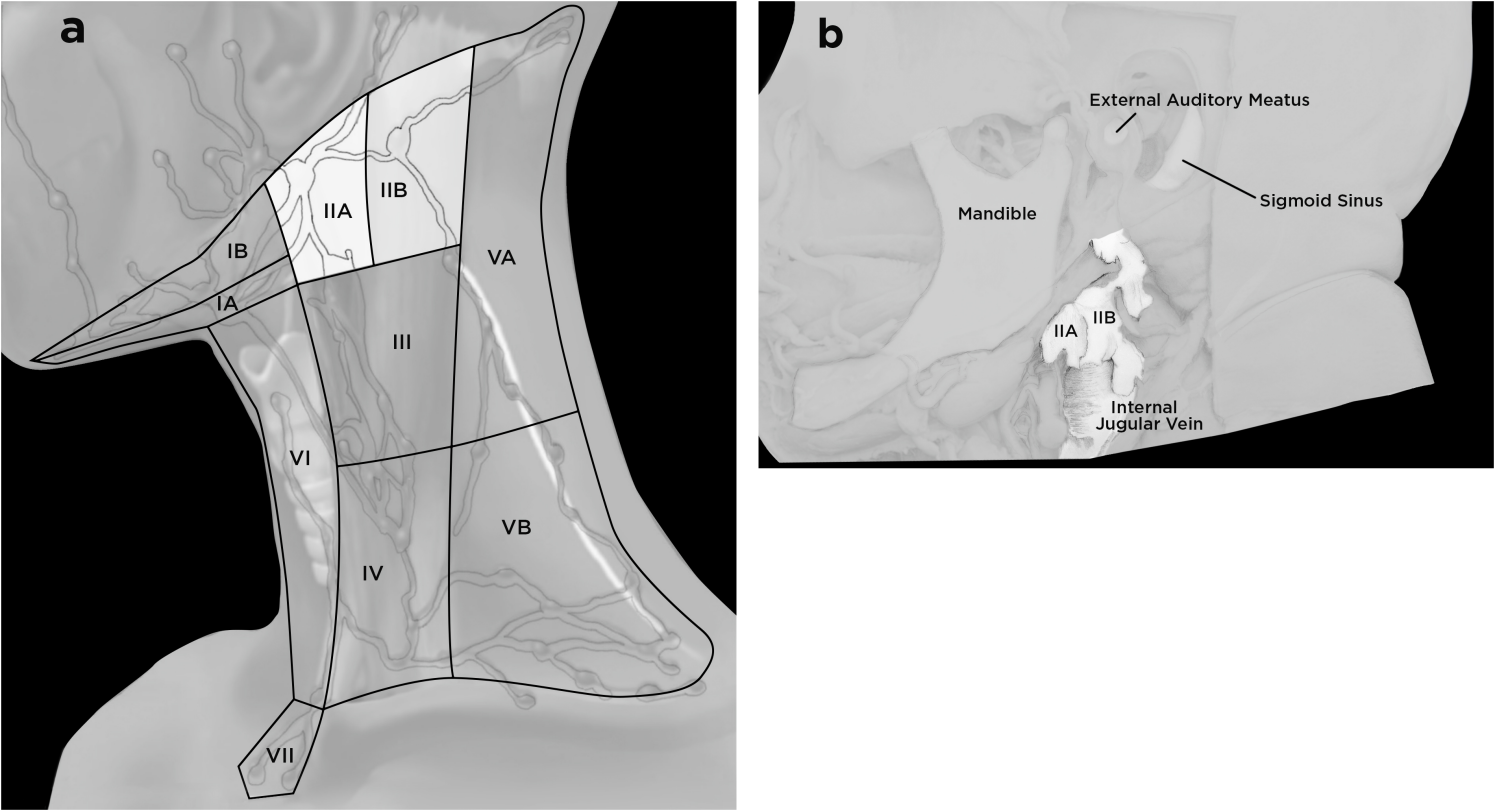
Cervical lymph nodes. **(A)** Lymph nodes in the neck can be divided into seven areas (sometimes referred to as “levels”): area II corresponds to the upper jugular/upper deep cervical nodes, which are the focus of our discussion. The image has been redrawn from reference 51, with permission. **(B)** In this image, the sternocleidomastoid muscle has been resected, and a window has been created at the skull base to show the location of the sigmoid sinus, which drains to the internal jugular vein (IJV). Area II can be subdivided into IIA, which include nodes that contact the IJV anteriorly and IIB nodes, which are posterior to the IJV. A fat plane separates the IIA and IIB areas. The image has been redrawn from reference 50; the original is in the public domain, Creative Commons CC.

Of the body’s hundreds of lymph nodes, almost half can be found in the head and neck. In gross dissection, cervical lymph nodes run along the internal jugular vein (IJV), deep to the sternocleidomastoid muscle (SCM), and can be divided into areas (sometimes known as “levels”): area II, corresponds to the upper jugular/upper deep cervical nodes; area III corresponds to the mid-jugular/middle deep cervical nodes; and area IV corresponds to the lower jugular/lower deep cervical nodes (**Figure 2**) (50). Area II can further be subdivided into IIA, consisting of nodes that contact the IJV directly, and IIB, consisting of nodes that lie posterior to the IJV and are separated from IIA by a fat plane (**Figure 2B**).

The dcLNs, comprising areas II, III, and IV, receive lymphatic drainage from multiple regions of the head and neck, including the scalp, face, oral cavity, nasal cavity, and notably from the CNS via the meningeal and nasopharyngeal lymphatic pathways (**Figure 2A**) (51). One animal study found that approximately 50% of CSF volume drains to dcLNs (43). *In vivo* evidence indicates that glymphatic drainage from human brain ends in dcLNs (52). These nodes ultimately drain into the subclavian vein (IJV), thence to the right heart. Due to their role in regional drainage, dcLNs are also common sites of metastasis for head and neck malignancies, with nodal involvement and extracapsular spread used as prognostic indicators (53, 54). Ultimately, as we discuss in the next section, dcLNs are unique in that they can both receive antigens from the CNS and mount an immune response that can affect the brain.

## 3 Immune cell infiltration into the CNS

In the setting of injury and disease in the CNS, antigens from the brain, various metabolites, and damage-associated molecular patterns (DAMPs) trigger an innate and adaptive immune response both within the CNS and in peripheral lymphoid organs (43). When human serum albumin was injected into the CSF of rats, it led to antigen-specific antibody production within cervical lymph nodes (55). Cervical lymph nodes are thus proposed to serve as hubs connecting CNS inflammation with peripheral immune responses. Many therapies focus on immune cell infiltration into the CNS and target tissue migration to minimize immune-mediated damage (22).

Research indicates that peripheral immune cells enter the CNS. Dendritic cells (DCs), a professional antigen-presenting cell (APC) type, have been shown to migrate into the CNS along several chemokine gradients (22). B-cell receptor (BCR) repertoire sequencing in MS patients has shown that clonally expanded B cells in the CNS likely have origins in dcLNs due to degree of clonal expansion (7). Studies of neutrophils in mice have shown that some neutrophils and monocytes in the mLVs come from cranial and vertebral marrow, not the blood, before subsequently infiltrating the brain’s parenchyma (56).

T cells can also infiltrate the CNS. For T cells to traverse the blood-brain barrier (BBB), the BBB must express inflammation-induced adhesion molecules that mediate transendothelial migration, a process that increases in pathological conditions (57). Moreover, during neuroinflammatory states, the BBB becomes more permissive, facilitating migration to the brain parenchyma. In mice that entirely lack mLVs, T-cell infiltration into the parenchyma is reduced (58). Although T cell homing to the CNS is increased during pathological conditions, T cells may also surveil the CNS even in non-inflammatory states. Blockade of T cell migration to the CNS using alpha-4 integrin biologics resulted in rare cases of JC virus reactivation in MS patients, implying a role for T-cell trafficking in CNS immune surveillance (59, 60).

Recent work in animals has shown that immune cell trafficking from the periphery to the CNS is linked to certain anatomical routes. Antigens from the posterior component of the eye, for instance, also drain to dcLNs (40). As a result, these antigens can induce immune responses in the brain involving CD4^+^ T cells, B cells, and locally produced antibodies (40, 61–64). In contrast, superficial cervical lymph nodes (scLNs), which are excluded from the dcLN system delineated above, do not receive antigens from CNS as readily, and drainage to scLNs is not enough to mount a CNS immune response in most circumstances (40, 51). These findings highlight that immune cell entry into the CNS is not random. Rather, it depends on specific anatomical structures, with dcLNs acting as a central gateway for antigen sampling and immune cell response.

## 4 Immune cell and antigen drainage from the CNS

A bidirectional connection between the immune and nervous system has long been recognized, but progress in understanding the mechanisms of CSF drainage from the brain has begun to elucidate how mLVs influence peripheral immune responses. In many disease states, innate and adaptive immune cells accumulate in the CNS (22). Some of these cells—including DCs, B and T cells, monocytes, neutrophils, and parenchymal border macrophages (PBMs)—can exit the CNS via the mLVs (22). These cells can subsequently enter the peripheral lymphoid organs and precipitate an autoreactive immune response (43). DCs—one of the major cells that can coordinate immunity in the periphery—are guided by chemokine gradients and lymphatic signaling molecules to dcLNs, where they can prime or induce T cell tolerance in the lymph node (22).

It is unclear whether DCs are local resident cells in the CNS or whether they infiltrate from the periphery into the CNS; however, research using bone marrow-derived, tracer-labeled DCs injected into brain parenchyma has demonstrated that DCs can drain to dcLNs (65). Importantly, the brain parenchyma contains very few conventional DCs under normal conditions. The cells with DC markers in the CNS could represent true dendritic cells, transformed microglia, or cells from the periphery (12, 65). B cells in the meninges can also drain from the mLVs to the cervical lymph nodes via the transverse and sigmoid sinuses, where they can trigger anti-inflammatory and pro-inflammatory responses in the periphery (66, 67). CD4^+^ T cells, which promote progression of neuroimmunologic diseases like multiple sclerosis (MS), similarly drain via the mLVs to the dcLNs (68). Research has shown that mLV injury results in the accumulation of T lymphocytes in the meninges (69). Changes in chemokines can also affect T cell drainage (62). Monocytes are another cell type that has been shown to leave the brain and migrate to the dcLNs via the cribriform plate (70). Neutrophils in the CNS exit via initial transport to mLVs before drainage to the neck; specific mechanisms for the bidirectional movement of neutrophils are active areas of investigation (43). Finally, PBMs have been shown to affect arterial pulsatility and therefore CSF flow (71).

In states of homeostasis, autoantigens which are involved in self-recognition and peripheral immune tolerance, drain from the CNS through the mLVs to the dcLNs. In the dcLNs, antigen-presenting cells (APCs) such as DCs, present peptides in major histocompatibility complex (MHC) II to CD4^+^ T cells, triggering a pathogenic or tolerogenic T cell response via induction of FoxP3^+^ T-regulatory cells depending on the context (72). Immune tolerance is the desired consequence of this interaction, but unrestrained autoreactive T cell activation can occur when homeostasis is altered through such processes as inflammation, infection, or injury. Research has shown that macrophages in the CNS and B cells in the dcLNs each present CNS antigens to T cells in distinct ways—which can shift the immune response from promoting tolerance to potentially increasing the risk for autoimmunity (72). Studies further suggest that the delivery of CNS antigens by migratory DCs to lymph node-resident DCs, which live in lymph nodes, can drive lymphocytic proliferation and alter adaptive immune cells in the periphery (22).

## 5 Sequelae of dysfunctional CNS clearance: selected diseases

Disturbance of the glymphatic system can contribute to disease in several ways. *β*-amyloid, reactive oxygen species (ROS), and tau protein, among other molecules, leave the CNS through the glymphatic system (73). While low to moderate ROS may have favorable effects (a phenomenon known as hormesis), excessive levels can result in apoptosis and cellular death (74). Traumatic brain injury (TBI) has been shown to reduce glymphatic pathway function by 60% for at least a month post-injury (75). Reduced tau clearance after injury sets the stage for accumulation of that protein and perhaps higher likelihood of ensuing neurodegeneration (8, 76).

The mLVs are known to be involved in the drainage of metabolic byproducts, cellular debris, and cells. The “debris” may include *β*-amyloid proteins, tau proteins, α-synuclein, TDP (transactive response DNA-binding protein)-43, glutamate, as well as senescent astrocytes and red blood cells (RBCs) (43). The elimination of senescent astrocytes, for instance, is important for homeostasis during aging (43). Similarly, RBCs from hemorrhagic stroke drain via the mLVs—a process crucial for preventing negative sequelae of infarction, hemorrhage, or both (43, 77, 78).

Failure of clearance from the extracellular space is implicated in the pathogenesis and progression of Alzheimer disease (AD), small-vessel disease, stroke, diabetes, migraine, microinfarcts, glaucoma, and glioblastoma (75, 79–87). That diverse neurological diseases share a common theme of impaired lymphatic drainage is analogous to how vascular dysfunction underlies cardiovascular diseases. The extracellular accumulation of *β*-amyloid is known to be part of the pathogenesis of AD and cerebral amyloid angiopathy (CAA); enhancing the function of mLVs through vascular endothelial growth factor C (VEGFC) injections has been shown to promote lymphangiogenesis and solute clearance—a promising, albeit still underexplored therapeutic avenue (43, 88, 89).

Several other proteins and metabolites play major roles in neurodegeneration. Tau protein accumulation, which is implicated in progressive supranuclear palsy, corticobasal degeneration, various frontotemporal dementias, and argyrophilic grain disease, among other tauopathies, is cleared by mLVs (43). The role of mLVs in tau clearance highlights the lymphatic system as a target for treatment in these diseases as well (43). Similarly, α-synuclein, which is involved in the pathogenesis of Parkinson disease (PD), has been shown to accumulate in the brain after cervical lymphatic ligation, with associated glial activation, inflammatory response, and dopaminergic neuronal loss (43). Two other molecules, TDP-43 and glutamate, are cleared by the mLVs. Both are implicated in the pathogenesis of amyotrophic lateral sclerosis (ALS).

Acute and chronic peripheral inflammation are key extrinsic factors that can modulate CNS clearance. In rodents, systemic administration of lipopolysaccharide impaired clearance and interstitial bulk flow of *β*-amyloid in a way that could either precipitate or promote AD pathology (90).

A table summarizing various brain solutes that drain from the glymphatic system through the mLVs to the dcLNs is shown below (**Table 1**) (32, 43). A host of regulators can affect mLVs based on animal studies, including pharmacological and physical interventions (**Table 1**) (43). Various pathologies can impact mLV morphology just as changes in the mLVs can affect disease (58). Thus, modulating mLV clearance to aid CNS drainage holds promise as a future therapeutic approach.

**Table 1:** Brain solutes cleared via mLVs and key pharmacological and physical regulators of mLV function (43).

Category	Specific Agents
Brain-derived solutes cleared via mLVs	Oligomeric α-syn, β-amyloid, tau proteins, transactive response (TAR) DNA-binding protein 43 (TDP-43), glutamate, cellular debris
Pharmacological Regulators of mLV function	Vascular endothelial growth factor C (VEGFC), borneol (BO), Yoda1, vitamin D, yuanzhi powder (YZP), DNase I
Physical Regulators of mLV function	Repetitive transcranial magnetic stimulation (rTMS), electrical stimulation, transcranial near infrared (NIR), acoustic regulation

Note that regulators have not been systematically examined in large patient populations to date.

To understand the rationale behind various attempts, mainly in animal models, at pharmacologic regulation of mLV function, a basic familiarity with lymphatic endothelial cell (LEC) development beginning at the genetic level is useful. These endothelial cells develop as a result of VEGFC signaling through the VEGF receptor 3 (VEGFR3); initial lymphatic development in embryos depends on this interaction (91). Expression of Prospero-related homeobox 1 (Prox 1), a DNA-homeobox transcription factor, facilitates expression of VEGFR3, while VEGFC activity is potentiated by collagen and calcium binding endothelial growth factor 1 (CCBE1) and the zinc-dependent protease ADAMTS3 (a disintegrin-like metalloproteinase with thrombospondin type 1 motif, 3). Absence of CCBE1, VEGFC, and VEGFR3 is associated with impaired development of mLVs.

Maintenance of LECs, as well as formation of lymphatic valves—particularly in basal mLVs of the CNS—relies on multiple intermediaries in pathways that are implicated in both mLV injury and repair (42). Selected therapeutic agents and the rationale for their use, based primarily on an animal study, include exogenous VEGFC; borneol (BO), which crosses the blood–brain barrier and increases VEGF expression upstream of Forkhead box O1 (FOXO1), a factor that facilitates LEC maintenance; Yoda1, an agonist of the mechanosensitive ion channel Piezo1, which is associated with increased VEGFC expression vitamin D, which is thought to increase vascular endothelial (VE) cadherin, a calcium-dependent adhesion protein involved in maintaining lymphatic vessel integrity; Yuanzhi powder (YZP), which may aid the correct polarization of aquaporin channels at astrocytic endfeet; and DNase I, which may promote degradation of neutrophil extracellular traps (NETs) within mLVs after cerebral hemorrhage (43). Numerous other potential therapeutic agents are under investigation.

Sample physical regulators in Table 1 are thought to modulate or stimulate mLV drainage. Transcranial near-infrared light may act at the level of mitochondrial cytochrome c oxidase (CcO), a photoreceptor whose activation has been associated with lymphatic endothelial cell (LEC) proliferation.

### 5.1 The role of sleep in disease

Sleep is a critical regulator of brain waste clearance. Abnormalities in sleep are highly prevalent in patients with neurodegenerative diseases and can also serve as early predictors of phenotypic conversion in some diseases. The largest risk factor for protein aggregation is age, as sleep quality decreases with age (92). People over 60 do not spend as much time in slow-wave sleep, at which time the ISF volume fraction increases (93).

There is a bidirectional relationship between poor sleep and neurodegeneration. For instance, the buildup of α-synuclein in the hypothalamus leads to the loss of orexin-containing neurons, which are implicated in promoting wakefulness (94). As disturbed sleep worsens, a vicious cycle perpetuates, with both impaired clearance and increased accumulation of brain waste.

### 5.2 Disturbed mLVs and downstream effects

The disruption of mLVs profoundly alters CNS immune balance and tissue health. In a mouse model of Alzheimer disease, ablation of mLVs induced a sustained pro-inflammatory state characterized by microgliosis and worsened amyloid deposition, whereas enhancing lymphatic drainage with VEGFC improved immune clearance and behavioral outcomes (95). In a related animal study, mLV ablation altered glial gene expression, reduced the population of mature oligodendrocytes, and, during demyelination, induced an immunosuppressive state that delayed remyelination (96). Under normal conditions, mLVs continually drain into the cervical lymph nodes. When this pathway is interrupted or ablated, T and B cell populations in both the meninges and cervical lymph nodes increase, and microglia in adjacent white matter adopt a phenotype that amplifies oxidative stress (96). As a result, mature oligodendrocytes are lost and myelin depletes. In mice with demyelination, immune suppression is characterized by decreased levels of cytokines and chemokines, delayed replenishment of oligodendrocytes, and increased axonal loss. All the above—to emphasize, in an animal study—suggests a two-part derailment: in part immunosuppression, in part autoimmunity (96).

Dysfunction in mLV signaling is increasingly recognized as a driver of various neurological diseases. VEGFC, a growth factor that regulates meningeal lymphatic growth (see **Table 1**), is reduced in the CSF of patients with MS (96); the reduction, which is seen especially in the post-relapse period in relapsing-remitting disease, implies that impaired VEGFC signaling may underlie mLV dysfunction in human disease. Chronic mLV failure has been linked to an array of brain disorders, because of both impaired metabolite and debris drainage as well as dyscoordination between peripheral and CNS immune cells. Compromised mLV function can ultimately exacerbate neurodegeneration and intensify neuroinflammatory responses through a variety of mechanisms (**Table 2**) (43).

**Table 2:** Reported roles of meningeal lymphatic drainage across CNS conditions (32, 43).

Disease category	Specific diseases	Role of meningeal lymphatic drainage
Neurodegeneration	Alzheimer disease (AD) (animal and human studies) (81, 97–99), Parkinson disease (PD) (animal and human study) (100, 101), Progressive Supranuclear Palsy (PSP) and other tauopathies (human study) (102), TAR DNA-binding protein-related (e.g., TDP-43) disease, including traumatic brain injury (TBI) and other TDP-43-associated diseases (animal studies) (103, 104)	Clearance of aging cells or cellular debris, clearance of β-amyloid, α-synuclein, phosphorylated tau, TDP-43, *inter alia*
CNS hemorrhage	Intraparenchymal (both superficial and deep), intraventricular, subarachnoid, and subdural blood (animal studies) (77, 105–107)	Clearance of red blood cell (RBC) debris and potentiation of immune-related reaction to injury
CNS ischemia	Various stroke types (large and small vessel) (animal and human studies) (108–111)	Clearance of cellular debris especially from interstitial fluid (ISF)
CNS neoplasm	Various types (animal and human studies) (112–114)	Potentiation of immune-related tumor response
CNS neuroinflammation	Bacterial, viral, and other inflammatory etiologies (animal studies) (115–118)	Initiation and perpetuation of immune-related response; clearance of virus from CNS

### 5.3 Immune function and modulation of dcLNs

In an animal study, resection of the dcLNs has been shown to increase meningeal T cells, likely reflecting impaired drainage and clearance (39). In mouse models of PD, dcLNs enlarge because of α-synuclein draining from the CSF and the presence of activated macrophages (100). Removal of dcLNs in a mouse model of chronic-relapsing experimental autoimmune encephalomyelitis (EAE) reduced relapse frequency but not acute phase severity, suggesting that dcLNs initiate CNS antigen-specific immune responses, including intermolecular epitope spreading, during relapses (119). More recently, surgical excision of cervical lymph nodes increased phosphorylated tau accumulation, worsening AD-like pathology in animal models (102).

In MS, fine-needle aspiration of dcLNs found increased Epstein-Barr virus (EBV) DNA (120). Dysfunction in dcLN drainage may also play a role in the development of AD, PD, and TBI. The above findings are relevant to current therapeutics, because they suggest that therapies affecting the periphery can have strong effects on the CNS, with dcLNs potentially acting as sentinel sites for CNS pathology in humans.

Fundamentally, the role of dcLNs extends beyond drainage to that of a critical interface that can be modulated. In 2024, researchers determined that the nasopharyngeal lymphatics (NPLP) regress in aging mice, but dcLNs do not (121). Since dcLNs can still respond to pharmacological stimulation, such as phenylephrine or sodium nitroprusside, CSF drainage can be regulated because of the effect on dcLNs. Therefore, these nodes highlight a therapeutic target for augmenting waste clearance (especially if and when nasopharyngeal drainage declines). The persistence of dcLN responsiveness even when other lymphatic routes fail with aging means these deep nodes could serve as a “last resort” therapeutic target when other interventions are no longer effective.

## 6 Imaging of cervical lymph nodes and the glymphatic system

Since dcLNs are implicitly involved in immune response, noninvasive visualization of dcLNs could aid in diagnosing and monitoring CNS inflammation. However, these applications remain exploratory in the clinical setting at present. Various modern head and neck imaging techniques can be employed to investigate dcLNs, including computed tomography (CT) scans, magnetic resonance imaging (MRI), and positron emission tomography (PET)-CT. While most imaging protocols initially evolved for oncologic indications, we will discuss potential adaptations for neuroinflammatory conditions.

MRI provides superior soft tissue contrast and may be more sensitive than CT for revealing subtle node changes in neuroinflammatory disease. Routine MR sequences include 2D and 3D options with T1- and T2-weighted imaging, performed with and without fat saturation (FS). While the utility of various techniques is still debated, one study examining axillary lymph nodes found that T2-weighted fast spin echo (FSE) images showed the highest number of lymph nodes, while T1 fast field echo (FFE) had the most anatomical information (122). Ultrasound (US) is less favorable due to limitations in visualizing deeper nodes but can sometimes be used in children to reduce radiation exposure (50).

In practice, distinguishing a lymph node can be difficult, since many types of structures can appear similarly (50). After a lymph node is identified, it must be characterized as normal or abnormal. Normal lymph nodes are kidney-shaped with a central fatty hilum; changes in this fat can indicate nodal pathology (50). If a level I or II cervical node exceeds 15 mm, it is also considered abnormally enlarged; while size was previously the main way to distinguish a pathologic node, size is not always a clear indicator of pathology (50, 123). Instead, the most important findings are configuration, homogeneity, enhancement, loss of the normal fatty hilum, and preservation of surrounding fat planes (50).

Imaging techniques may be able to identify subtle asymmetries or changes in nodal architecture even before overt enlargement. If the lymph node is rounded, has no fatty center, or is enlarged, it should be flagged. However, lymph node size is not always positively correlated with disease severity in the context of inflammation. In MS, cervical lymph node diameter on MRI was inversely correlated with disease duration and disability progression, with node size diminishing during acute relapses (124). Quantitative T1 nodal “maps” can also reveal asymmetries. Particularly, T1 asymmetry of the lymph node cortex between affected and contralateral axilla can be ascertained by quantitative relaxation time mapping in breast cancer treatment-related lymphedema; the asymmetry increases with cancer stage (125). Ultimately, node measurements should always be considered in the context of scan technique and disease state.

An important advance in lymphatic imaging was the demonstration of glymphatic drainage to dcLNs using MR. One 3D T1-weighted *in-vivo* MR study showed evidence of glymphatic drainage from the brain to dcLNs, with cervical lymph node enhancement occurring at the same time as peak glymphatic enhancement (52). Similarly, 3D T2-FLAIR (fluid-attenuated, inversion recovery) MRI without contrast showed that dorsal and ventral mLVs can be visualized, in addition to their direct relationships with dcLNs (16). Another MRI study examined whether ligation of dcLNs affected CSF tracer dynamics, and found that CSF outflow into the nasal cavity was blocked, causing retention (126).

In addition to traditional MR, advanced techniques are increasingly being utilized to interrogate the glymphatic system *in vivo*. These include perivascular space assessment, DTI (diffusion tensor imaging) analysis along perivascular spaces (DTI-ALPS), arterial spin labeling, and chemical exchange saturation transfer, which can be used to evaluate the glymphatic system *in vivo* (127). Lower DTI-ALPS indices have been correlated with higher burdens of amyloid in AD, increased risk of dementia conversion in PD, and larger tumor volumes in patients with glioblastoma, brain metastases, and aggressive meningiomas (128–130). Glymphatic dysfunction as measured by DTI-ALPS and free water fraction are similar in MS and neuromyelitis optica spectrum disorder, indicating that neuroinflammatory processes, regardless of exact disease etiology, may impair glymphatic clearance in similar ways (131). Contrast-enhanced glymphatic MRI has shown that, after subarachnoid hemorrhage in humans, CSF is redistributed toward the ventricles and fluid transport in perivascular spaces is impaired, underscoring how vascular injury can disrupt glymphatic function (132). Emerging noninvasive imaging biomarkers for lymphatic function may eventually help to identify patients at increased risk for neurodegeneration, although clinical validation is still needed. If validated clinically, this development could shift the field toward earlier risk stratification and prevention.

The integration of conventional and advanced MRI protocols can allow for not only anatomical, but also functional and quantitative assessment of dcLNs and glymphatic system activity. Leveraging these approaches with CSF assays and immunophenotyping of nodal tissue can yield powerful biomarkers and help to develop a stronger understanding of neuroimmune dynamics.

The limitations of imaging are those not infrequently encountered in general clinical practice. Nodal anatomy is variable; imaging protocols must be standardized (and uniquely tailored to identify nodes); and artifactual findings are inevitable (133–135).

## 7 Sampling and the relevance of immunophenotyping

The deep cervical lymph nodes (dcLNs) could highlight immunologic processes occurring in the CNS, especially when CSF findings are inconclusive. Though lymph node study as a diagnostic or monitoring tool is still experimental, with unproven reproducibility in clinical settings, the avenue holds promise. Immunophenotyping of nodal tissue could identify unique immune cell populations that might not be evident in peripheral blood or CSF. A study in rats found that bone marrow-derived myeloid DCs injected into the CSF infiltrated the brain parenchyma and trafficked to the cervical lymph nodes in an EAE model, compared to controls—all of which is thought to then elicit both local (CNS) and systemic immune responses (136).

**Figure 3:**
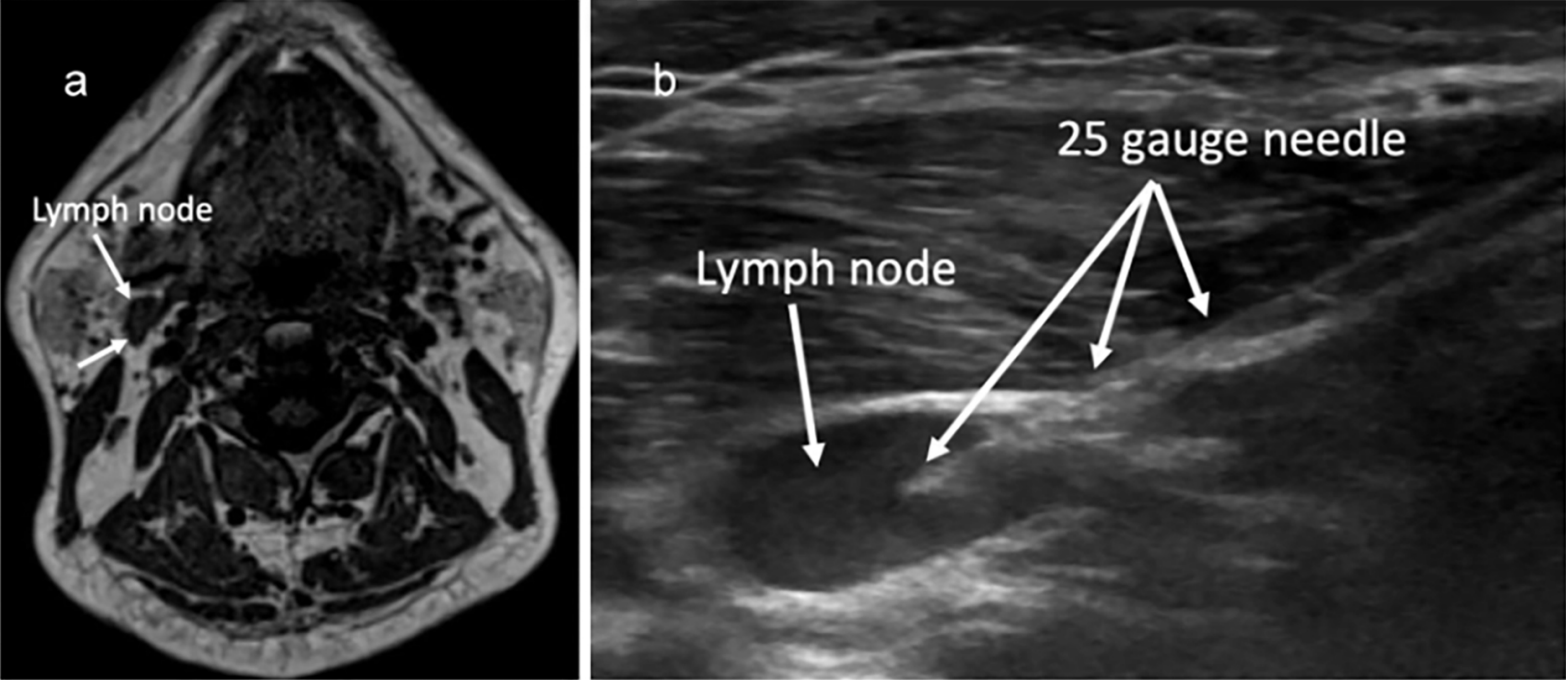
Axial MR image and ultrasound-guided fine-needle aspiration (FNA). **(A)** Arrows point to a deep cervical lymph node in the vicinity of the internal jugular vein (dcLN level IIA) in the right neck. This node is normal in size and safely targetable for FNA, which is routinely performed in clinical practice. **(B)** During FNA, after local anesthesia, a 24-gauge needle is advanced into a dcLN.

As dcLN groups IIA and IIB (see **Figure 2**) are the sites particularly relevant to CNS glymphatic drainage in humans, they are an optimal choice to assess. When imaging nodes, clinicians should note shape, enhancement patterns, hilum appearance, cortical thickness, and nodal architecture, as well as evidence of necrosis or calcification. After imaging with MR, percutaneous ultrasound-guided fine-needle aspiration (FNA) or core-needle biopsy can be used for sampling (**Figure 3**). FNA is often preferred because it is minimally invasive, relatively fast, and has a lower complication rate than other options. The sample consists of small tissue fragments and individual cells. The material from FNA sampling can then be used for cytology, flow cytometry, or molecular analysis. Flow cytometry may identify expanded or atypical lymphocyte clones, while immunohistochemistry can distinguish specific immune subsets and activation states. For tissue sampling and better diagnostic accuracy, however, either a core needle biopsy or examination of a whole dcLN is superior, albeit far more invasive.

Immunophenotyping can indicate subsets of T cells, B cells, monocytes, DCs, *inter alia*. An overlap in B-cell or T-cell receptor sequences between dcLN and CSF samples can show whether immune clones are spreading from the periphery into the CNS or *vice versa*. Unlike CSF sampling (e.g., of cytokines) which captures a single timepoint, dcLN immunophenotyping may reveal the dynamic trajectory of an immune response. As a result, for example, clinicians could distinguish resolving inflammation from progressive autoimmunity and thereby select personalized and more precise therapies.

Histopathologic and immunophenotypic assessment has been conducted in MS patients (120, 137). The minimal amount of tissue required for definitive analysis might be debated; the first of the cited studies examined whole lymph nodes, whereas the second involved FNA. Fine-needle samples may not suffice in characterizing the nature of an adaptive immune response originating in dcLNs. Nevertheless, the value of combined histologic and immunophenotypic lymph node analysis lies in the ability to detect changes in T- and B-cell populations that occur particularly in the context of MS relapse—and presumably in other forms of active neuroinflammation—along with alterations in nodal germinal center (GC) reactions, which reflect antigen-specific T- and B-cell interactions. Within GCs, B-cell hypermutation, as occurs in MS, is dependent on follicular T helper (Tfh) cells and an increased ratio of Tfh to T follicular regulatory (Tfr) cells, as was learned from whole-node analysis. Notably, even fine-needle aspiration (FNA) samples subjected to single-cell RNA sequencing or combined transcriptomic and epitope indexing were sufficient to identify disease-specific changes in clonal B-cell populations, including those of GC origin.

### 7.1 Clinical applications and translational proof of concept

In patients with inflammatory CNS disorders, sampling dcLNs can complement standard CSF testing (and other testing they may undergo). For instance, when oligoclonal bands or cytology are not conclusive, clinicians could use dcLNs to identify antigen-”experienced” B cells or other activated antigen-presenting cells, although such applications remain preliminary. Such findings could ultimately strengthen a diagnosis and clarify how certain conditions affect both the CNS and peripheral lymphatic system.

Serial imaging and immunophenotyping of dcLNs may one day facilitate disease-course tracking. On MRI, one might observe node enhancement or changes in size that may indicate times of intensified glymphatic flow or flares of CNS inflammation (16, 52, 124). The addition of immunophenotyping can reveal, in detail, how the character of immune populations within the nodes shifts in the context of therapy. Altogether, these tools allow clinicians to witness both the architecture and the choreography of immune activity over time.

Yet the lymphatic landscape of the neck is more than a series of checkpoints. As previously discussed, early animal experiments suggest that enhancing lymphatic function through agents like VEGFC, or physical interventions such as electrical or acoustic stimulation, may amplify the brain’s natural capacity to clear metabolic debris (43, 86, 87). In the future, treatments in humans might aim to guide the immune response in these nodes, perhaps by introducing specialized DCs or other selected immune cells to help restore balance. These possibilities remain experimental, so immense caution is necessary. Removing cervical nodes interrupts critical drainage pathways and may disrupt the intercommunication between the CNS and the periphery, leading to deleterious effects beyond the accumulation of harmful proteins in the brain.

A diagnostic pathway should be straightforward and, ideally, elegantly ordered. First, we might identify lymph nodes of interest using dedicated MRI followed by ultrasound. Next, ultrasound-guided fine-needle aspiration can allow for the procurement of cellular material for immunophenotyping. Each result must be interpreted in context alongside CSF analysis, blood tests, and imaging findings. By repeating these assessments during relapses or following therapy adjustments, clinicians can follow the unfolding story of disease.

## 8 Discussion

### 8.1 Current research gaps, potential developments

Despite recent breakthroughs in understanding CNS-lymphatic interactions, several research gaps persist. One area of ongoing investigation is the anatomical variability and functional significance of alternate lymphatic drainage routes, particularly the nasopharyngeal lymphatic plexus (NPLP). While studies in mice and non-human primates highlight the NPLP’s involvement in CSF clearance, its relevance in human physiology remains uncertain (41, 48). The extent to which NPLP regression with age impacts CNS immune dynamics is also not yet elucidated. Recent work demonstrates that the NPLP undergoes involution in aging mice, whereas dcLNs persist as structures that may be of particular anatomical and functional relevance (121).

There is also considerable debate regarding the potential of surgical and pharmacological interventions to restore or augment lymphatic clearance in neurodegenerative and neuroinflammatory diseases. For example, a hypothesis has been proposed regarding the use of deep cervical lymphovenous bypass surgery to enhance intracranial lymphatic drainage in the treatment of PD (138). Controlled clinical trials are lacking, and the risk–benefit ratio of such interventions remains to be determined.

Another promising yet underexplored avenue is immune modulation at the nodal level. The directed migration of DCs from the CNS to dcLNs depends on chemokine signaling and can be experimentally blocked in animal models, illustrating that immune cell egress is not a passive process, but rather can be manipulated (65). Delivering antigen-specific DCs to cervical lymph nodes could perhaps alter pathogenic immune responses in diseases like MS without needing broad immunosuppression (22). However, the application of such targeted cellular therapies depends on careful preclinical and clinical validation.

Imaging and molecular phenotyping have the potential to further accelerate clinical progress. Novel MRI techniques, like DTI-ALPS (diffusion tensor imaging along perivascular spaces), perivascular space assessment, and quantitative MRI mapping, could allow for a dynamic assessment of glymphatic and lymphatic pathway function *in vivo* (16, 52, 127). Pairing these readouts with serial node sampling and immune repertoire profiling in clinical studies could allow for the evaluation of disease-modifying interventions at both the anatomical and cellular levels (128–132).

Lastly, safety must be considered. While lymph node excision has shown negative consequences in animal models—including accumulation of pathogenic proteins and worsening of neurodegeneration (102)—minimally invasive sampling (fine-needle aspiration or core biopsy) provides actionable clinical information with lower risk (50). Standardized imaging follow-up and careful patient monitoring for complications, such as neck swelling or new neurological symptoms, will be essential components of any interventional trial. The research community must also resist the temptation to simply translate animal findings directly to humans; we need human-first studies that establish whether findings in non-human animals are similarly present in human CNS-lymphatic interactions.

### 8.2 Future directions

Deep cervical lymph nodes stand at the nexus of central and peripheral immune surveillance. They uniquely reflect and regulate immune dynamics in the brain. More than passive drainage points, dcLNs are now recognized as active modulators, sentinel sites, and potential therapeutic targets in a wide range of neurological diseases.

Advances in imaging, like dedicated high-resolution MRI, have rendered these nodes and the drainage pathways leading to them much more accessible for *in vivo* examination (16, 52). The ability to sample and immunophenotype lymph node tissue—complementing CSF and blood biomarker analysis—could offer new methods to diagnose, monitor, and manage a variety of neuroinflammatory and neurodegenerative disorders. While these applications remain exploratory, the rationale for integrating these analyses into clinical practice has become quite compelling (43, 69, 88, 89, 96).

Multiple questions remain. It is essential to clarify the effects of aging, sleep, and peripheral inflammation on lymphatic flow and node function (28, 31, 36, 92–94). The reversibility of node dysfunction, efficacy of possible interventions (whether surgical or molecular), and long-term safety must all be addressed. Despite these ongoing questions, the field’s progress in recognizing the nervous and immune systems as interconnected rather than independent domains should not be understated.

We advocate for three immediate priorities to advance this field. First, we recommend establishing multicenter consortia to collect standardized imaging and immunophenotyping data from dcLNs across neurological diseases. Once there is compelling evidence, we advise launching early-phase human trials of lymphatic enhancement strategies (VEGFC, sleep optimization, physical interventions) with rigorous endpoints perhaps including serial dcLN sampling. Finally, we suggest integrating dcLN assessment into existing clinical trials of neuroinflammatory disease in general. Pending further experimental and human validation, these steps have the potential to transform the CNS-lymphatic axis from a novel and exciting biological discovery into a practical clinical tool.
